# Plasma Sterilization Effectively Reduces Bacterial Contamination in Dental Unit Waterlines

**DOI:** 10.1155/2019/5720204

**Published:** 2019-07-30

**Authors:** Sarocha Noopan, Phattranit Unchui, Supitcha Techotinnakorn, Ruchanee Salingcarnboriboon Ampornaramveth

**Affiliations:** Research Unit on Oral Microbiology and Immunology, Department of Microbiology, Faculty of Dentistry, Chulalongkorn University, Bangkok, Thailand

## Abstract

**Objective:**

To investigate the effectiveness of plasma sterilization in reducing bacterial contamination and controlling biofilms in dental unit waterlines.

**Materials and Methods:**

Ten identical dental chair units (DCUs) were used. Five DCUs were installed with an automated plasma sterilization system (PSS) and the other five were kept as nontreated controls (CTL). Water flushed from the airotor line served as the output water of the dental unit waterlines (DUWLs). Water samples were collected at the beginning and on a weekly basis for 4 months. Water was analyzed for bacterial contamination (CFU/mL). Scanning electron microscopy (SEM) was used to investigate the amount of biofilm in the waterlines. Biofilm viability was assessed by 3-(4,5-dimethylthiazol-2-yl)-2,5-diphenyl-2H-tetrazolium bromide (MTT) assays. All statistical analyses were performed using the Mann–Whitney *U* test. A value of *p* < 0.05 was considered significant.

**Results:**

The DCU output water was found to be heavily contaminated with bacteria. Plasma sterilization effectively reduced bacterial contamination from an average of 212 CFU/mL to 8 CFU/mL. During the entire period of 4 months, the level remained below 500 CFU/mL, the standard level recommended by the Centers for Disease Control and Prevention (CDC) of the USA. The reduction in the bacterial count was significant compared with the CTL group (*p* < 0.05). Plasma sterilization could not eradicate the existing biofilms in the waterlines, and it did reduce biofilm mass and viability. Moreover, treatment with plasma sterilization did not induce a change in the composition of microorganisms, as analyzed by Gram staining.

**Conclusion:**

Plasma sterilization, which is part of electrochemically activated water, effectively reduces bacterial contamination and reduces biofilms in dental unit waterlines.

## 1. Introduction

Dental chair units make use of water as a coolant for high-speed rotary dental instruments and for washing the mouth during the dental procedure. The input water of a dental chair unit can be derived from different sources: either directly from municipal water or from a separate water reservoir. Once it enters the dental chair unit, the water will be flushed through the complex small tubing system to supply the airotor handpiece and air-water syringe. The dental chair unit output water has been reported to be highly contaminated by microorganisms [1]. Predominant bacteria species found in dental unit waterlines (DUWLs) are Gram-negative aerobic heterotrophic environmental species including *Pseudomonas* species, particularly *Pseudomonas aeruginosa*, and *Legionella* species, particularly *Legionella pneumophila*, and nontuberculosis mycobacterial species [2–4]. The microorganisms in the tubing system form biofilms which continuously release microorganisms that contaminate output water. Contamination of DUWLs thus poses a serious concern in infection control in dental practice.

The main obstacle to eliminate effectively microorganisms in DUWLs is the formation of biofilm that aggravates resistance to a wide range of disinfection methods. Currently, several methods are proposed to reduce bacterial contamination in DUWLs. The most popular method is disinfecting with chemical disinfectants. Commonly used products are peracetic acid, ethylenediaminetetraacetic acid, tetrasodium salt, sodium hypochlorite, chlorhexidine gluconate, povidone iodine, hydrogen peroxide, and chlorine dioxide [5]. Previous studies indicated that chemical disinfectants including peracetic acid [6], hydrogen peroxide [7], *N*-halamine [8], D-amino acid [9], povidone iodine [10], and nanosilver [11] significantly reduced the bacterial count in DUWLs. However, most of them could not totally eradicate biofilm in long-term use [12]. Some disinfectants (e.g., chlorine compounds and acetic acid) even caused damage to the water pipes [13, 14]. Most of these disinfectants could only be used as shock treatment due to their harmfulness to the patients and need more compliance from staff in dental clinic.

An alternative approach using plasma sterilization technology for disinfecting DUWLs is widely used in the Korean dental profession. The machine produces positive and negative charges, so-called plasma ions, and this disinfects the water system. The plasma particles are generated by high-voltage electricity that split passing water into the reactive oxygen species OH^−^, H_2_O_2_, and O_3_. The plasma system utilized in this study is a platinum catalyst and produces plasma with low temperature in water, and as a result, H_2_O is resolved into H^+^, O^−^, and O_3_ and form OH^−^, HOCl, and H_2_O_2_. These plasma particles have a bactericidal effect due to their strong oxidizing properties. Despite the effectiveness claimed by the company, very few studies report the efficacy of plasma sterilization in decontaminating biofilms in dental unit water lines [15].

The purpose of this study was to investigate the effectiveness of plasma sterilization on reducing bacterial contamination and controlling biofilms in dental unit waterlines.

## 2. Materials and Methods

### 2.1. Sample Collection

Ten identical dental chair units (DCUs) at the Faculty of Dentistry, Chulalongkorn University, were selected for the study. The DCUs were divided into 2 groups: (1) plasma sterilization system (PSS) and (2) nontreatment control (CTL). The chairs of the PSS group were equipped with a plasma sterilization system (DPS-110bi, Dentozone, Seoul) which resulted in a continuous generation of plasma ions into the DUWLs.

At the start of the experiment, water from 2 sources was collected from each unit to assess baseline bacterial contamination. These two sources were as follows: the cup-filled water was kept as input water which was obtained directly from the main water inlet of the building and water flushed from the airotor line served as DUWL output water. The water samples were collected weekly for 4 months to analyze bacterial contamination of the dental unit output water.

### 2.2. Laboratory Investigation

After collection, the water samples were transferred directly to the laboratory and kept at 4°C until plate count was done. In order to break up clumped bacterial biofilms, the water samples were sonicated for 10 seconds before serial dilution and they were subsequently plated onto R2A agar (HiMedia, Mumbai, India) and incubated at 36°C for 3–7 days. The number of bacterial colonies was counted and expressed as colonies forming unit per mL (CFU/mL). Colony formation and Gram staining were performed to characterize bacteria found in the water before and after treatment with PSS. Gram staining was performed as follows: the colonies were smeared onto a glass slide and fixed with heating using a flame. The smear was then stained with crystal violet, iodine, washed with alcohol, and counterstained with safranin.

Scanning electron microscopy was used to investigate whether plasma sterilization could eliminate biofilm formation on the waterlines. At the end of the experimental period (4 months), the tubes of the DUWLs were collected, fixed in 2.5% glutaraldehyde for 24 hours, and washed with 1% PBS before sectioning into small pieces. The specimens were serially dehydrated, critical point dried, gold sputter coated, and examined by using scanning electron microscopy (SEM) (FEI Quanta 250, OR).

MTT assays were used to determine the biofilm viability. The tubes from DUWLs were soaked in 100 *μ*L of 0.5 mg/mL MTT solution (Affymetrix, CA) and incubated for 3 hours at 37°C. The MTT solution was then removed, and 100 *μ*L DMSO was added and incubated for 15 minutes at room temperature. The OD value was measured at a wavelength of 570 nm.

All data were analyzed and compared using the Mann–Whitney *U* test. A *p* value of 0.05 was considered statistically significant. The Ethics Committee of the Faculty of Dentistry, Chulalongkorn University, Thailand, has approved the study to be carried out according to the protocol and informed permission dated and/or amended as follows in compliance with the ICH/GCP (HREC-DCU 2018-006).

## 3. Results

**Figure 1:**
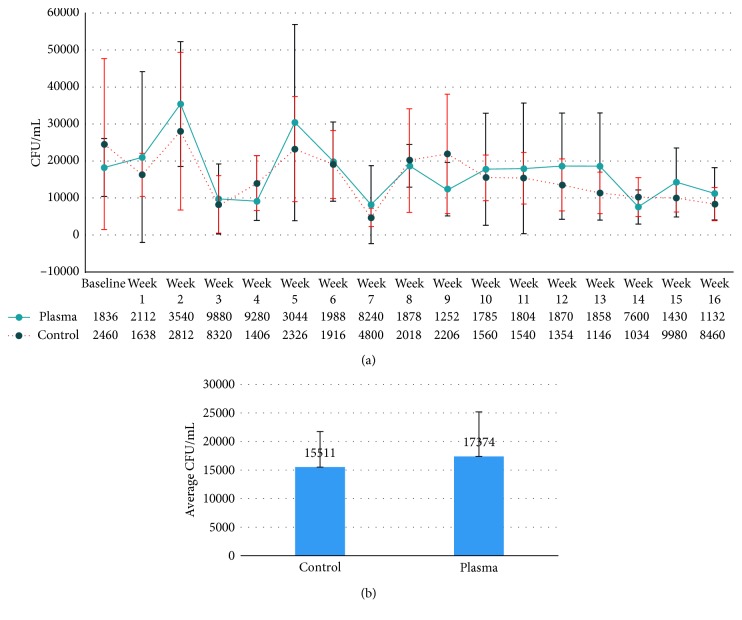
(a) Total bacterial count from dental chair unit input water. Each line indicates the average CFU/mL at different time points. The blue line represents the average CFU/mL from input water of the PSS group. The red line represents the average CFU/mL from input water of the control group. The average number of the bacterial count in CFU/mL is presented in the table below the chart. (b) The average CFU/mL of the entire period of the experiment. The bacterial count of input water of the two groups is not significantly different.

Dental chair unit input water was heavily contaminated with bacteria ranging from an average of 4,800 to 30,440 CFUs/mL. There was not a significant difference in the bacterial count of input water between the PSS and the nontreatment control group (Figures 1(a) and 1(b)). Throughout the entire period of 4 months, the dental unit output water was found to be heavily contaminated with bacteria. The maximum bacterial counts were as high as an average of 35,400 CFUs/mL.

**Figure 2:**
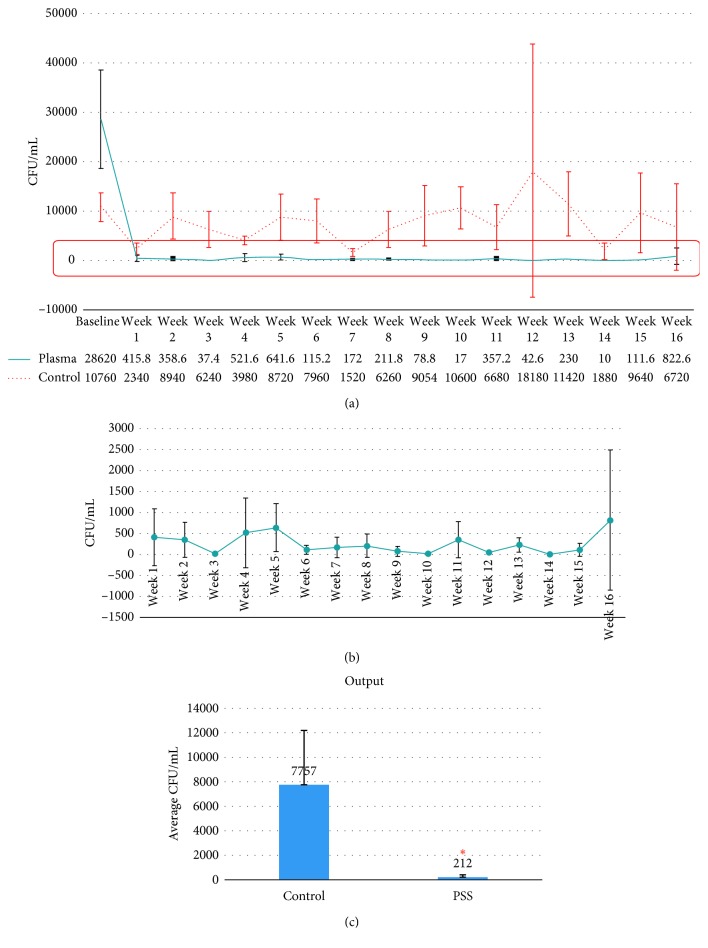
(a) Total bacterial count from dental chair unit output water (from airotor line). Each line indicates the average CFU/mL at the different time points. The blue line represents the average CFU/mL from output water of the PSS group. The orange line represents output water of the control group. The number of bacterial counts (CFU/mL) is presented in the table below the chart. (b) Magnified view of the area shown in the red box of (a). PSS effectively reduced the bacterial number and was found to be within the standard level recommended by CDC of less than 500 CFU/mL (the red dotted line). (c) The average CFU/mL throughout the period of experiment. The average bacterial count observed in the PSS group is significantly lower than that in the controlled one. ^*∗*^Statistically significant difference at *p* ≤ 0.05.

After the first week of plasma sterilization, the number of bacteria was dramatically reduced and maintained at the same level until the end of the experiment at week 16 (Figures 2(a) and 2(b)). The average CFU/mL of bacteria recovered from airotor line of PSS group was 212 CFU/mL, which was significantly lower than the control group (*p* < 0.05) (Figure 2(c)).

**Figure 3:**
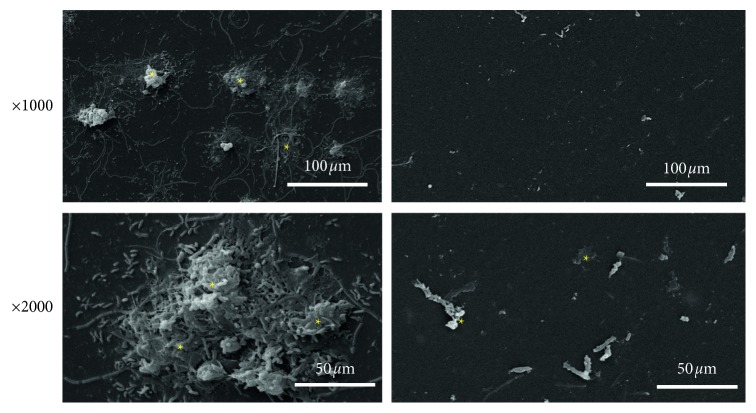
Scanning electron microscopy revealed the presence of biofilms that covered the inner surface of the waterlines. Bacterial biofilms were reduced in the PSS group (right panel) compared with the control group (left panel). ^*∗*^Bacterial biofilm. The lower panels show a higher magnification (×2000).

**Figure 4:**
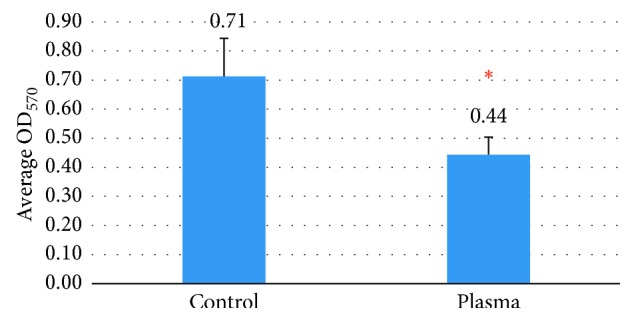
MTT assay indicated the viability of biofilm in the watertubes. PSS reduced the amount of viable biofilms in the waterlines. ^*∗*^Statistically significant difference at *p* ≤ 0.05.

Scanning electron microscopy revealed the presence of bacterial biofilms covering the inner surface of the waterlines in the control group (Figure 3). The biofilm mass was strongly reduced in the PSS group (Figure 3). In line with the reduced amount of biofilm, MTT analysis revealed a significantly reduced viability of these samples (Figure 4).

**Figure 5:**
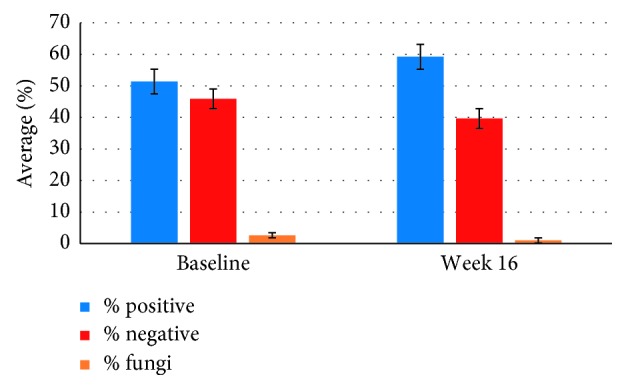
Gram staining of bacterial colonies recovered from output water before and after PSS treatment. Gram positive (blue bar); Gram negative (red bar); and fungi (orange bar). PSS did not induce a shift in types of microorganisms.

According to the results obtained from the Gram staining, the PSS did not induce a selective shift in types of microorganisms. The colonies found in output water at baseline and 16 weeks after PSS installation still have the same Gram staining profiles. The ratio of Gram positive, Gram negative bacteria, and fungi were not different compared to the baseline from week 16 (Figure 5).

## 4. Discussion

Our results demonstrated that plasma sterilization significantly reduced bacterial contamination in dental unit waterline output water when applied as continuous treatment. The average CFUs/mL of bacteria recovered from DUWL of the plasma sterilization group was less than 500 CFU/mL. This value is within the standard level recommended by the American Dental Association (ADA). SEM analysis revealed that after 4 months of PSS treatment the bacterial biofilms inside the waterlines were reduced. The MTT assay also indicated that the system effectively decreased biofilm mass as reflected by a reduction in viability. According to previous studies reported by Marais and Brozel [12], the efficacy of electrochemically activated (ECA) water was demonstrated; ECA markedly reduced the number of bacterial colonies (<1 CFU/mL) and also eliminated the biofilm more completely than a conventional disinfectant (0.5% sodium hypochlorite solution). Treatment with ECA resulted in a smooth and clean surface of the waterlines. Meanwhile, the biofilm in the sodium hypochlorite-treated group proved to be cracked. Our study proposes a clinical application of the plasma sterilization system for the reduction of bacterial contamination in dental unit waterlines.

There are several approaches to control bacterial contamination in dental unit waterlines including both nonchemical and chemical methods. However, most of these methods provide uncertain effects on controlling bacterial biofilms in the complex tubing system of DUWLs. Therefore, the effectiveness of the plasma sterilization system in reducing both bacterial count and biofilm mass appears to be an attractive way to control biofilms inside the complex tubing system of dental chair units.

Currently, for decontamination processes, plasma ions are used as an energy reservoir to activate a variety of chemicals present in or added to the solutions. Likewise, in our study, by using the electrochemically activated principle, the plasma particles are positively and negatively charged and produced by high-voltage electricity. These charges will split water molecules into the reactive oxygen species (ROS) OH^−^, H_2_O_2_, and *O*_3_^−^. In this way, PSS generates strong oxidants which provide bactericidal effects so that the exopolymer matrix that coats the biofilms collapses due to hydroxyl radicals. Then, the bacterial colonies inside are exposed to other oxidative agents and subsequently killed [12]. This could explain the marked reduction in bacterial counts and the elimination of the biofilm as noted in our study. The plasma technology, and especially hydrogen peroxide gas plasma sterilization, has been developed to provide a broad-spectrum bactericidal effect. Due to their instability, plasma ions tend to release an extra amount of free energy which drives it toward an equilibrium stage. Thus, it is difficult to measure the amount of plasma ion quantitatively. In this study, we qualitatively confirmed the presence of hydroxyl radical in output water by the methylene blue dye test as reported by Satoh et al. [16]. Briefly, PSS water generated from the machine (at the machine outlet tube) was immediately dropped on the methylene blue dye test trip. Bleaching of methylene blue dye, due to the presence of hydroxyl radicals in a sample, was indicated by a discoloration from a dark blue color to an almost white color. A lack of bleaching indicated the absence of hydroxyl radicals in the sample. The result confirmed the presence of OH^−^ in the PSS water.

According to the results obtained with the Gram staining, treatment with PSS did not induce a change in the composition of the microorganisms. However, this approach does not give information on the presence and/or absence of specific groups of bacteria. Further studies are needed to analyze the exact composition of microorganisms found before and after treatment with PSS.

A sincere concern of the use of the PSS system is its safety. Plasma ions are very strong oxidizing agents that can oxidize biological molecules found in living organisms. Although there is no evidence that confirms or disclaims safety of the system, the system is likely to be safe for the following reasons. First, the plasma particles are produced by an electrical charge applied to water and do not consist of any toxic chemical compound. Second, the patients are only exposed to a very small volume of water from DUWLs. Thus, the limited exposure of small quantities of free radicals should not be hazardous. There are some experiments that support safety issue of electrochemically activated solution which shares the same principle as our PSS. A single-dose and a 28-day repeated oral dose of super oxidized water in rats did not produce any serious adverse effects [17]. Furthermore, a test with human cells demonstrated no oxidative damage on nucleic acids or the cells themselves [18]. Nevertheless, experiments with animals and cell cultures are still needed to prove the safe use of the plasma sterilization system.

## 5. Conclusions

The plasma sterilization system, which is part of electrochemically activated water, effectively reduced bacterial contamination and biofilms in dental unit waterlines. This study, therefore, proposes PSS as a treatment to decontaminate dental unit waterlines. In addition, it may be applied to control bacterial biofilms in other medical devices.

## Data Availability

The datasets used in this study were supplied by Ruchanee S Ampornaramveth, DDS, PhD. Requests for access to these data should be made to Ruchanee S Ampornaramveth (ruchanee.a@chula.ac.th).
